# A step towards a holistic assessment of soil degradation in Europe: Coupling on-site erosion with sediment transfer and carbon fluxes

**DOI:** 10.1016/j.envres.2017.11.009

**Published:** 2018-02

**Authors:** P. Borrelli, K. Van Oost, K. Meusburger, C. Alewell, E. Lugato, P. Panagos

**Affiliations:** a European Commission, Joint Research Centre, Directorate for Sustainable Resources, Ispra, Italy; b Environmental Geosciences, University of Basel, Switzerland; c TECLIM - Georges Lemaître Centre for Earth and Climate Research, Université Catholique de Louvain, Louvain-la-Neuve BE 1348, Belgium

## Abstract

Soil degradation due to erosion is connected to two serious environmental impacts: (i) on-site soil loss and (ii) off-site effects of sediment transfer through the landscape. The potential impact of soil erosion processes on biogeochemical cycles has received increasing attention in the last two decades. Properly designed modelling assumptions on effective soil loss are a key pre-requisite to improve our understanding of the magnitude of nutrients that are mobilized through soil erosion and the resultant effects. The aim of this study is to quantify the potential spatial displacement and transport of soil sediments due to water erosion at European scale. We computed long-term averages of annual soil loss and deposition rates by means of the extensively tested spatially distributed WaTEM/SEDEM model. Our findings indicate that soil loss from Europe in the riverine systems is about 15% of the estimated gross on-site erosion. The estimated sediment yield totals 0.164 ± 0.013 Pg yr^−1^ (which corresponds to 4.62 ± 0.37 Mg ha^−1^ yr^−1^ in the erosion area). The greatest amount of gross on-site erosion as well as soil loss to rivers occurs in the agricultural land (93.5%). By contrast, forestland and other semi-natural vegetation areas experience an overall surplus of sediments which is driven by a re-deposition of sediments eroded from agricultural land. Combining the predicted soil loss rates with the European soil organic carbon (SOC) stock, we estimate a SOC displacement by water erosion of 14.5 Tg yr^−1^. The SOC potentially transferred to the riverine system equals to 2.2 Tg yr^−1^ (~15%). Integrated sediment delivery-biogeochemical models need to answer the question on how carbon mineralization during detachment and transport might be balanced or even off-set by carbon sequestration due to dynamic replacement and sediment burial.

## Highlights

• WaTEM/SEDEM was applied to simulate soil loss and deposition rates at European scale.
• Our findings indicate that soil loss in the riverine systems is about 15% of RUSLE2015 estimates.
• The estimated sediment yield in Europe totals 0.164 ± 0.013 Pg yr^−1^.
• We estimate a SOC displacement by water erosion in Europe of 14.5 Tg yr^−1^.

## 1 Introduction

The recognition of detrimental effects of soil erosion can be dated back to Classical Greek philosophers such as Plato and Aristotle (Runnels, 1995). In our days, erosion is known as one of the most critical forms of soil degradation and a major threat to agricultural soil productivity (FAO ITPS, 2015) and thus, in many regions of the world, to societal stability. Intensive farming practices significantly accelerate soil erosion rates (Zhao et al., 2013) up to about two orders of magnitude (Montgomery, 2007). The effects of soil erosion can be severe, not only on-site through land degradation and fertility loss but also causing serious off-site damage like eutrophication of waters, clogging of river beds or damage to infrastructure.

On a global scale, estimates of soil loss by water erosion in agricultural areas range from 23.7 to 120 Pg yr^−1^ (Doetterl et al., 2012), with the soil loss due to inter-rill and rill processes recently estimated at about 17 Pg yr^−1^ (Borrelli et al., 2017). Starting with the pioneering study of Stallard (1998), the soil science community has paid increasing attention to the potential impact that such vast displacement of soil may have on climate through erosion-induced changes on the carbon biogeochemical cycle (Quinton et al., 2010). Soils represent the largest terrestrial reservoir of carbon globally, only exceeded by the oceans and the fossil carbon in the lithosphere and they are estimated to store up to three times the organic carbon present in the atmosphere (2413 ± 37 Pg C to a depth of 2 m) (Lal, 2003). The decade old discussion on erosion and carbon content has not yet brought to a unanimous opinion clearly indicating if soil erosion increases or decreases CO_2_ emissions through enhanced mineralization versus sediment burial (Lal, 2004, Van Oost et al., 2007). It may depend on the type of land-use and management practices if soil must be perceived as a sink or source of atmospheric CO_2_ (FAO ITPS, 2015).

Soil carbon sequestration through improved land management is seen as a great opportunity by both scientists and decision-makers (Paustian et al., 2016). During the COP21 in Paris the French authorities launched the 4‰ initiative which rests on the hypothesis that a slight increase in net soil carbon storage would represent a considerable carbon sink potential (Minasny et al., 2017). They suggested that a 4‰ annual growth rate of soil carbon stock would stop the present increase in atmospheric CO_2_ (4 per 1000 initiative, 2016). The initiative was presented as a win-win situation where improved land management and carbon sequestration could enhance both the quality of agricultural soils and the soil carbon storage. This would have the potential to reduce soil erosion and soil degradation thereby improving soil productivity and surface water quality.

Various attempts have been made to estimate soil displacement and induced lateral C (carbon) fluxes (Van Oost et al., 2007, Lal and Pimentel, 2008, Nadeu et al., 2015, among others). While these approaches vary in complexity, the scale has generally been limited to small catchment and regional levels. State-of-the-art large-scale applications mainly rest on a combined use of the RUSLE (Renard et al., 1997) model to estimate on-site soil loss and biogeochemical models for the lateral carbon fluxes occurring with the sediment transportation such as RothC (Chappell et al., 2015) or CENTURY (Borrelli et al., 2016, Lugato et al., 2016). Since RUSLE only provides gross erosion estimates, net soil erosion estimates and data-driven assumptions are needed to assess the amount of transported SOC, decreasing uncertainty on the predicted carbon fluxes (Van Oost et al., 2007, Lugato et al., 2016).

In order to understand the significance of omitting soil erosion from soil organic carbon cycling schemes (Chappell et al., 2015), today's challenge is to reduce the current modelling assumptions on soil erosion/deposition dynamics and move towards more mechanistic approaches. In a context where process-based physical models and the availability of input data are not yet mature enough for large-scale applications (Jetten et al., 2003, De Vente and Poesen, 2005), simple and physically plausible empirical methods for predicting soil erosion such as RUSLE can provide reasonably accurate estimates. However, since RUSLE-type models only provide gross erosion, the integration of a further module in the RUSLE scheme to estimate the sediment yield from the modelled hillslopes is needed. Excluding simple sediment delivery ratio (SDR) obtained from regression analysis (USDA, 1975), a better approach would be to model the sediment dynamics as a function of land use and topographical conditions. In this context, the spatially distributed sediment delivery model WaTEM/SEDEM (Van Oost et al., 2000, Van Rompaey et al., 2001) which has been widely tested in Europe (Alatorre et al., 2010, Alatorre et al., 2012, Bakker et al., 2008, Van Rompaey et al., 2005, among others) represents a valid option. Moreover, since WaTEM/SEDEM uses the RUSLE parameters to incorporate the impact of soil and cover to estimate of net erosion and deposition, it is fully compatible with the new assessment of soil loss by water erosion in Europe (RUSLE) (Panagos et al., 2015).

In this study, we present quantitative estimates of net soil erosion and deposition rates at European scale. We use a high-resolution (25 × 25 m) application of the spatially distributed sediment delivery model WaTEM/SEDEM. The latest state-of-the-art data for modelling soil erosion in Europe are employed. Besides the net sediment fluxes, we also present preliminary approximations of potential carbon loss and dynamic replacement in Europe.

## 2 Materials and methods

### 2.1 Study Area

**Fig. 1:**
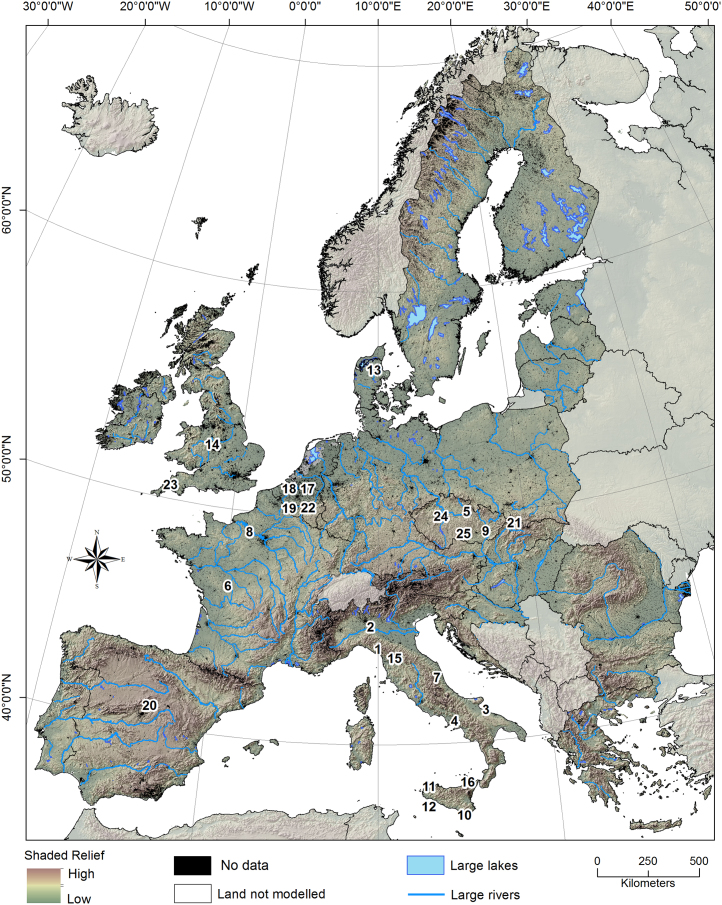
Study area. The shaded relief highlights the modelled area (European Union member countries (EU28)). The numbers indicate the distribution of the catchments used for the calibration of WaTEM/SEDEM (1 Mignano; 2 Molato; 3 Locone; 4 Letino; 5 Vrchlice; 6 Le Louroux; 7 Scandarella; 8 Austreberthe; 9 Bilovice; 10 Disueri; 11 Prizzi; 12 Gammauta; 13 Gelbaek; 14 Kyre Pool; 15 Santa Luce; 16 Ancipa; 17 Hammeveld2; 18 Hammeveld1; 19 Ganspoel; 20 Pareja; 21 Brzezowa; 22 Kinderveld; 23 Old Mill Reservoir; 24 Nemcice reservoir; 25 Hamry reservoir).

Our modelling area covers about 3.86 million km^2^, corresponding to the erodible land of the European Union member countries (EU-28) as described by the land use / land cover map CORINE (Coordination of Information on the Environment) for the reference year 2006 (EEA-European Environmental Agency, 2016) (Fig. 1). Areas such as built-up, inland water bodies, wetlands, rocky surfaces and beaches were excluded. The resulting modelling area amounts to ~86% of the EU-28. Agricultural land covers a total surface area of 1.74 million km^2^ (45%), out of which 1.19 million km^2^ is arable land. The remaining land is covered by forest (44%) and other semi-natural vegetation (11%).

### 2.2 WaTEM/SEDEM model

The long-term annual rates of soil loss, sediment transfer and deposition were modelled with WaTEM/SEDEM (Van Oost et al., 2000, Van Rompaey et al., 2001). The model has been extensively employed to estimate net fluxes of sediments across landscape- (Lieskovský and Kenderessy, 2014, Quijano et al., 2016, among others), catchment- and regional-scale level (Verstraeten et al., 2002, Van Rompaey et al., 2001, Alatorre et al., 2010, among others). To the best of our knowledge, this study represents the first application at the continental scale.

WaTEM/SEDEM is a spatially explicit sediment delivery model involving two components. In the first stage, the soil loss potential is computed according to the multi-parameter scheme of the Revised Universal Soil Loss Equation (RUSLE) (Eq. (1)).

(1) $$\mathrm{SL}=R\cdot K{\cdot \mathrm{LS}}_{2D}\cdot C\cdot P$$

where SL is the mean soil loss (Mg ha^−1^ yr^−1^) which is the product of the rainfall intensity factor R (MJ mm ha^−^^1^ h^−^^1^ yr^−1^), the soil erodibility factor K (Mg ha h ha^−1^ MJ^−1^ mm^−1^), the two-dimensional slope and slope-length factor ${\mathrm{LS}}_{2D}$ (Desmet and Govers, 1996), the cover-management factor C (dimensionless) and the conservation support practice factor P (dimensionless).

In the second step, the displaced soil amount (gross erosion) is routed downslope across each pixel from hillslopes to the riverine systems according to the transport capacity (TC in Mg yr^−1^) (Eq. (2)), computed on the base of topography and land cover.

(2) $$\mathrm{TC}=\mathrm{ktc}\cdot E_{\mathrm{PR}}=\mathrm{ktc}\cdot R\cdot K\cdot \left({\mathrm{LS}}_{2D}-4.1\cdot S_{\mathrm{IR}}\right)$$

where TC is the transport capacity (Mg ha^−^^1^ yr^−1^), ktc (m) is the transport capacity coefficient, R, K, ${\mathrm{LS}}_{2D}$ are the aforementioned RUSLE input factors and $S_{\mathrm{IR}}$ (m m^−1^) (Eq. (3)) is the inter‐rill slope gradient computed based on Govers and Poesen (1988) (Eq. (3)):

(3) $$S_{\mathrm{IR}}=6.8\cdot S_{g}^{0.8}$$

where $S_{g}$ represent the slope gradient (m m^−1^).

For a more comprehensive description of the model components we refer to Van Oost et al. (2000) and Van Rompaey et al. (2001).

### 2.3 Model parameterization and calibration

To run WaTEM/SEDEM we employed the RUSLE parameters (R-, K-, C-, P-factor) recently developed by the Joint Research Centre in collaboration with several European scientists (Panagos et al., 2015). Since topography plays a central role in the model, a high-resolution (25 m) digital elevation model (DEM) was employed. The RUSLE parameters were resampled to 25 m through a nearest neighbor resampling algorithm to obtain a set of gridded layers spatially consistent.

To optimize the WaTEM/SEDEM simulations across the large modelling area, the calibration of the ktc coefficients, reflecting the vegetation component in the transport capacity, was conducted considering large ranges of values (${\mathrm{ktc}}_{\mathrm{low}}$ range 0–0.5, in steps of 0.05; ${\mathrm{ktc}}_{\mathrm{high}}$ range 20–600, in steps of 20). In addition, a range of different thresholds to define the upslope contributing area (Ac) was used (50, 100, 150 and 250 ha).

For the calibration of the model, a set of 24 catchments well distributed across Europe were employed. The catchment areas range from 2.5 to 245 km^2^. For each catchment ~1300 model runs were performed to simulate the sediment yield for each possible combination of ${\mathrm{ktc}}_{\min}$, ${\mathrm{ktc}}_{\mathrm{high}}$ and Ac. Subsequently, the model efficiency (ME, Eq. (4)) proposed by Nash and Sutcliffe (1970) was computed to evaluate the overall prediction capacity of each combination of parameters. Finally, the Generalized Likelihood Uncertainty Estimation (GLUE) methodology (Beven and Binley, 1992) was applied to represent the prediction uncertainty of our model. The deterministic model prediction is given by the median of the cumulative distribution function (CDF) (Blasone et al., 2008) while the associated uncertainty was selected at the 5% and 95% confidence level (Beven and Binley, 2014).

(4) $$\mathrm{ME}=1-\frac{{\sum_{i=1}^{n}\left({\mathrm{SY}}_{\mathrm{obs}}-{\mathrm{SY}}_{\mathrm{pred}}\right)}^{2}}{{\sum_{i=1}^{n}\left({\mathrm{SY}}_{\mathrm{obs}}-{\mathrm{SY}}_{\mathrm{mean}}\right)}^{2}}$$

where n refers to the number of observation, ${\mathrm{SY}}_{\mathrm{obs}}$ is the observed value of sediment yield, ${\mathrm{SY}}_{\mathrm{pred}}$ is the predicted value of sediment yield and ${\mathrm{SY}}_{\mathrm{mean}}$ is the mean value of the observed sediment yield. ME ranges from -∞ to 1. Values closer to 1 represent a higher model efficiency.

### 2.4 Detachment of SOC by erosion

The soil loss and deposition rates modelled with WaTEM/SEDEM were used to quantify the soil carbon detached by erosion ($C_{\mathrm{loss}}$) (Eq. (5)) in the European Union member countries (EU-28) agricultural soils:

(5) $$C_{\mathrm{loss}}=\mathrm{SOC}\cdot \left(\mathrm{SL}/(\mathrm{BD}\cdot \mathrm{SD}\cdot 100)\right)$$

where SOC is the soil organic carbon content of the spatially explicit topsoil layer (Mg ha^−1^ in the 0–30 cm) computed for Europe (1 km grid cell resolution) (Lugato et al., 2016), SL is the net soil loss estimated by WaTEM/SEDEM (Mg ha^−1^ yr^−1^), BD is the bulk density (Mg m^3^) derived from the topsoil physical properties map at European scale (500 m grid cell resolution) (Ballabio et al., 2016) and SD is the depth of the surface layer (30 cm).

## 3 Results and discussions

### 3.1 Model calibration

We employed long-term sedimentation records of twenty-four semi-natural and agricultural catchments to calibrate the WaTEM/SEDEM. A first calibration attempt was carried out considering all possible sets of available catchments to determine the best fit. The highest model efficiency (ME) derived from the best-parameter fit is 0.38. Although modest, this ME is in line with values observed by other WaTEM/SEDEM applications (Van Rompaey et al., 2003a, Van Rompaey et al., 2005, Feng et al., 2010, Quijano et al., 2016).

Using this calibration, an overall underestimation of ~8% in the predicted sediment yield was found. As also observed by Van Rompaey et al. (2005), when performing a global calibration using a uniform transport capacity factor for all catchments, the accuracy of WaTEM/SEDEM tends to be rather low. This is particularly true when semi-natural mountain catchments of the Mediterranean region are included in the calibration process (Van Rompaey et al., 2003a). The solid discharge in these catchments is often dominated by geomorphic processes that RUSLE-based models do not take into account, e.g., landslides, mudflows, gullying and river bank erosion (De Vente et al., 2006). For example, according to Borrelli et al. (2014) net loss of soil due to rill- and inter-rill erosion processes in sandstone-dominated intermountain catchments in central Italy appears to be responsible for only about 5–10% of the total sediment yield.

**Fig. 2:**
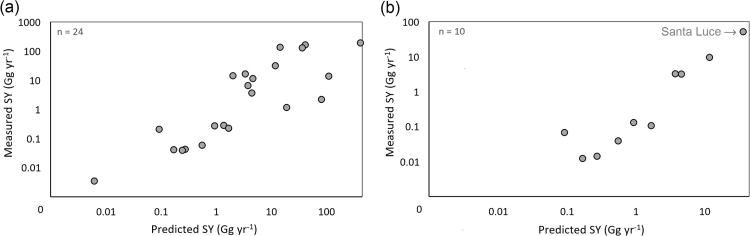
Predicted versus observed sediment yield (Gr yr^−1^) for the preliminary (a) and final (b) calibration using only the agricultural-dominated catchments (agricultural land > 65%).

A further calibration considering only the agricultural catchments (arable land > 65%; n = 10) showed a remarkable increase of the predictive capacity of the model (Fig. 2). Although the coefficient used for the final calibration (Fig. 2b) may seem to be affected by the extreme values of the Italian reservoir of Santa Luce, retesting the correlation without this catchment confirmed an equally high prediction capacity of the model (R^2^ = 0.98). In this second calibration, we observed an overall overestimation of the predicted suspended sediment yield with a difference of 10.5% between measured and predicted values. This is mostly driven by the conspicuous overestimation of sediment yield (~40%) in the largest catchment of the dataset (Santa Luce about 189 km^2^). The remaining nine catchments used for the second calibration show underestimations of the modelled sediment yield, which is consistent with the aforementioned inability of the model to account for the effect of other geomorphic processes.

Accordingly, for the final calibration only the ten catchments with more than 65% of arable land were employed. Following the Generalized Likelihood Uncertainty Estimation (GLUE) the optimal set of parameters for the median confidence level (ME = 0.89) is ${\mathrm{ktc}}_{\max}$ of 20 m, ${\mathrm{ktc}}_{\min}$ = 10 m and an upslope contributing area of 150 ha.

### 3.2 Soil erosion

The application of the WaTEM/SEDEM model provided spatially explicit estimation on the potential annual average soil displacement in the European Union member countries for the reference year 2010.

**Fig. 3:**
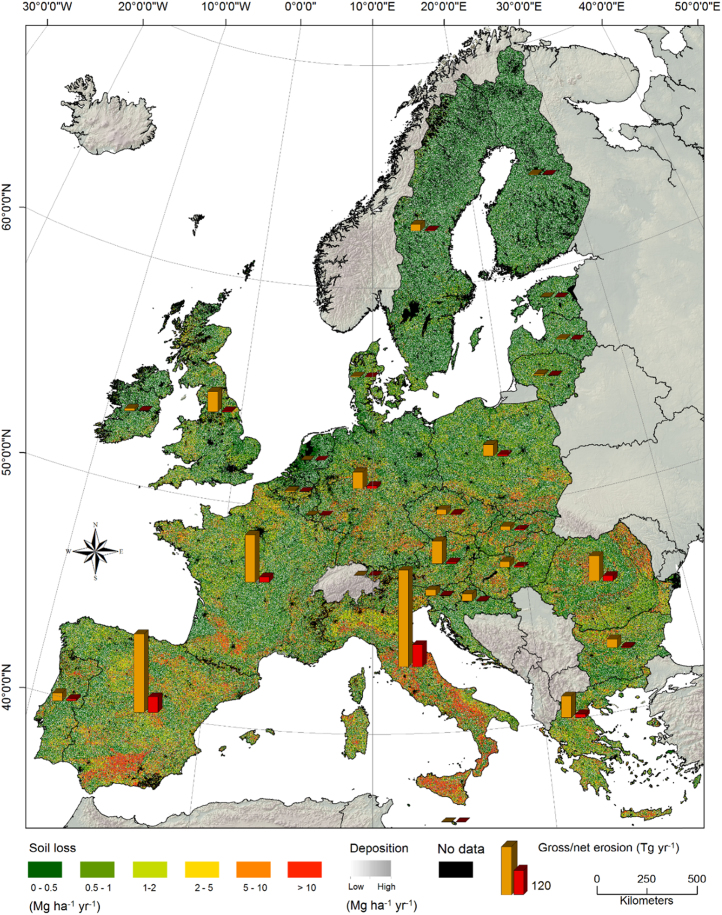
Estimated annual average soil loss and deposition rate for the European Union based on WaTEM/SEDEM. The vertical bars show the annual gross (orange) and net (red) soil losses in each country. (For interpretation of the references to color in this figure legend, the reader is referred to the web version of this article.)

The spatial pattern of soil loss and deposition rates are illustrated in Fig. 3. The model outcomes were separated into seven severity classes of soil loss. Deposition is represented in greyscale to optimize the readability of the map and to avoid color mixing. Areas classified as having erosion are about 64% of the modelled area, whereas no erosion or deposition cover the remaining 36% of land. Approximately 80.9% of the land surface prone to erosion shows very low and low predictions of soil loss (classes 1–2). Intermediate values (classes 3–4) cover about 11.7% of the land, while the remaining 7.4% (ca. 17.3 million ha) (class 5) shows predicted values exceeding the generic tolerable soil loss threshold of 10 Mg ha^−1^ yr^−1^.

The modelling results confirm the regional pattern previously illustrated by the application of RUSLE models (Panagos et al., 2015). Overall, the erosion rates show a southwest–northeast (SW–NE) oriented decreasing gradient. The highest erosion rates are found in Italy along the Apennines and the surrounding hilly areas, southern Spain, South of France and Romania. Soil erosion hot-spots at the European mid-latitudes are mainly concentrated along the area between southern Germany and Slovakia. Further north, very low (class 1) to low (class 3) erosion rates dominate the landscapes. Areas anomalously identified as possible hot-spots by previous RUSLE application (Panagos et al., 2015) (i.e., Scotland and the Scandinavian mountains), show low soil loss values in our model. Additional descriptive statistics at country level are provided by the vertical bars inserted in Fig. 3.

The average area-specific soil loss considering only the areas prone to erosion is 4.62 ± 0.37 Mg ha^−1^ yr^−1^. The average sediment yield predicted by the model for Europe totals 0.164 ± 0.013 Pg yr^−1^. This value corresponds to the net soil loss, that is, the fraction of displaced soil that leaves the landscape through the riverine systems. With regard to the gross on-site erosion, the predicted annual average of total soil mobilization is consistent with the results of Panagos et al. (2016), equal to 1.07 Pg yr^−1^. The sediment delivery ratio (SDR), i.e., the ratio between sediment yield (SY) and gross erosion, indicates that the sediment routed down the hillslopes to the riverine system accounts for 15.3% of the total eroded soil.

Comparing soil erosion dynamics based on land use types, we observed a noteworthy decline in soil loss rates from agriculture to forestland and other semi-natural vegetation areas. The soil loss estimated on agricultural land (1 Pg yr^−1^) is about 40 times higher than that of forestland (0.024 Pg yr^−1^) and 20 times higher than that of other semi-natural vegetation areas (0.046 Pg yr^−1^). Soils characterized by low erosion rates mainly appear in non-agricultural lands. In these low soil loss classes, farming is absent or mostly carried out on flat surfaces with small slope gradients (< 1 degree). Despite the fact that forestland and other semi-natural vegetation areas cover about 44% and 14%, respectively, of the modelled land, they have, on average, the lowest soil loss with about 2.2% and 4.3% of the total estimates. By contrast, soil erosion classes 3–5 are typical for human-dominated lands. About 95% of the lands showing predicted values exceeding the generic tolerable soil loss threshold of 10 Mg ha^−1^ yr^−1^ are agricultural. Accordingly, in about 16.4 million ha of the European Union (EU-28) agricultural area the prolonged high soil loss rates may have triggered a progressive decrease of the soils’ ability to sustain vegetation and livestock.

From a sediment budget point of view, the agricultural soils show a deficit at the European level, i.e., the soil loss is larger than the amount of sediment re-deposited within this specific land use type. This can be quantified in 0.278 Pg yr^−1^. By contrast, forestland and other semi-natural vegetation areas show a surplus of sediments driven by both local low erosion rates and by trapping sediments eroded on the agricultural land. Here, a deposition of 0.108 Tg yr^−1^ and 0.006 Pg yr^−1^ for the forestland and other semi-natural vegetation was observed, respectively.

**Fig. 4:**
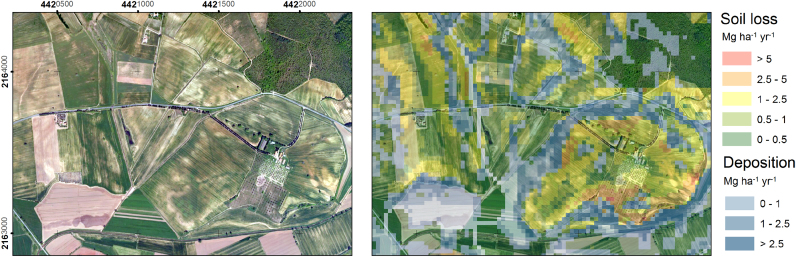
WaTEM/SEDEM results in hilly arable lands ongoing soil erosion and degradation processes in the Southern Tuscany (Magliano, 4421500 N − 2163500E).

A good example to illustrate the ability of WaTEM/SEDEM to predict soil erosion pattern is zooming into the highly-affected region of Tuscany (Fig. 4). Both, the severity of soil loss in the source areas (eroded-upper slopes and eroded water-ways) and toe-slope deposition areas are visible.

### 3.3 Carbon dynamics

Lugato et al. (2014) estimates the current SOC stock in the plough layer (0–30 cm) of European Union agricultural soils as 17.63 Pg. Combining the new estimates of soil loss with the SOC content provided by Lugato et al. (2014), we quantified for the European Union agricultural land a net SOC detachment of 14.5 Tg yr^−1^.

This straightforward estimation of net SOC detachment, similar to that previously carried out by Panagos et al. (2015), highlights the difference elapsing between the estimates of SOC displaced by water erosion considering gross and net erosion rates over a large and heterogeneous study area. According to our results, only about 15% of the SOC displacement estimated by Panagos et al. (2015) would effectively be lost in the riverine system (equal to ca. 2.2 Tg yr^−1^). The remaining ca. 12.3 Tg yr^−1^ would be redeposited across the landscape. Therefore, contrary to what was previously assumed in our previous study, to effectively erode 1% of the total 17.63 Pg of SOC in agricultural lands would be necessary 78 years instead of the previously assumed 12 years. The analysis on the soil loss and deposition fluxes for different land use cases showed that 72% (0.73 Pg yr^−1^) of the soil displaced in the agricultural land is redeposited within this area, while the rest is redeposited in other land uses. Thus, the considerable sedimentation in forests (0.108 Pg yr^−1^) and other semi-natural vegetation (0.006 Pg yr^−1^) may represent rather stable sinks for sediments and carbon. However we need to state, that these estimations do not consider possible mineralization of carbon during detachment and transport of sediments. Further analysis is required to quantify if our estimates of soil loss and deposition across Europe may enhance CO_2_ fluxes through mineralization or reduces them through burial. This aspect can be modelled integrating our new estimates into biogeochemistry models capable to comprehensively account for the multiple effects of soil erosion on lateral SOC fluxes (Nadeu et al., 2015, Lugato et al., 2016).

### 3.4 Scope, limitations and future directions

The simplistic approach in which WaTEM/SEDEM deals with landscape connectivity and the strong bond with the RUSLE components makes this model applicable over large areas. Although offering a parsimonious description of the processes, appreciable regional scale predictions of soil loss and sediment delivery from hillslopes to the riverine system were observed (Alatorre et al., 2012). Nevertheless, as observed by Van Rompaey et al. (2005) and confirmed by this study, the model prediction capacity decreases considerably when a unique coefficient of transport capacity is calibrated over multiple catchments (i.e., global calibration). With increasing complexity of the landscape, the straightforward calibration scheme of the model easily leaves up to 50% of the overall sediment yield variance unexplained (Feng et al., 2010, Van Rompaey et al., 2003a, Quijano et al., 2016). However, a better global calibration could be achieved reducing the heterogeneity of the set of calibration catchments (Van Rompaey et al., 2005).

In this study, we obtained an optimal calibration in considering agricultural-dominated catchments (agricultural land > 65%) separately. The rationale behind our decision to consider only the agricultural-dominated catchments is driven by i) the high contribution of these lands to the total soil loss in Europe (~60%, Panagos et al., 2015) and ii) the high importance of these areas in terms of their productivity and soil conservation potential from a land management point of view. Although this choice could have resulted in an overestimation of the transport capacity in the non-agricultural land, an overall ratio between ${\mathrm{ktc}}_{\max}$ ${\mathrm{ktc}}_{\min}$ (1:2.86) is in the range of typical values of 1:2.5–1:3.33 as reported in literature (Van Rompaey et al., 2003b; Verstraeten, 2006; Alatorre et al., 2010).

A better prediction capacity of the model in the agriculture-dominated catchments is consistent with the intrinsic structure of every RUSLE-based model, which has been conceived and developed using the statistical relationships observed in agricultural areas (Table 1).

**Table 1:** Descriptive statistics of the 24 catchments used for the model calibration.

Code no.	Name	Country	Area	Observed SSY	Arable land
			km^2^	Mg ha^−1^ yr^−1^	%
1	Mignano	Italy	87	12.8	39 (l)
2	Molato	Italy	81	10.1	48 (l)
3	Locone	Italy	245	1.7	62 (e)
4	Letino	Italy	13	0.5	8 (l)
5	Vrchlice	Czech Republic	97	0.46	60 (l)
6	Le Louroux	France	24	0.71	67 (e)
7	Scandarella	Italy	39	4.9	25 (l)
8	Austreberthe	France	206	0.16	61 (e)
9	Bilovice	Czech Republic	32	1.46	78 (e)
10	Disueri	Italy	189	16.8	57 (e)
11	Prizzi	Italy	21	5.7	77 (l)
12	Gammauta	Italy	91	1.6	52 (l)
13	Gelbaek	Denmark	12	0.8	95 (l)
14	Kyre Pool	UK	3	0.9	18 (l)
15	Santa Luce	Italy	40	9.2	70 (l)
16	Ancipa	Italy	50	5.6	0 (l)
17	Hammeveld2	Belgium	0.3	11.1	100 (l)
18	Hammeveld1	Belgium	0.3	5.9	100 (l)
19	Ganspoel	Belgium	1	4.8	87 (e)
20	Pareja	Spain	88	0.23	25 (l)
21	Brzezowa	Poland	5	0.01	5 (e)
22	Kinderveld	Belgium	3	3.78	82 (e)
24	Nemcice reservoir	Czech Republic	80	0.74	65 (e)
25	Hamry reservoir	Czech Republic	55	0.82	23 (l)

(e) estimated, (l) litterature.

However, as observed by Alatorre et al. (2010) the calibration of the transport capacity coefficients of WaTEM/SEDEM can be an important issue, independently from the good agreement between the predicted and measured sediment yield. To improve the predictive capacity of the model, spatially distributed calibration and validation processes are required (Vigiak et al., 2006; Alatorre et al., 2010). At European scale, this could be done following a stratified calibration procedure similar to the one proposed by Van Rompaey et al. (2005). The major European catchments (EEA-European Environmental Agency, 2017), could be divided into quasi-homogeneous units considering e.g., land use/ land cover patterns, topography, dimension and climate zone. Using a Monte Carlo calibration like the one proposed in this study individual best-parameter sets for each group of catchments could be computed. To do so, however, a dataset of long-term sedimentation records larger than the one available for this study (n = 24) would be required. Vanmaercke et al. (2011) recently analysed the sediment yield data from 507 reservoirs and 1287 gauging stations (n = 1794) across Europe, equal to ~30,000 catchment-year data. Such a comprehensive database would facilitate a more effective stratified calibration of WaTEM/SEDEM. Further improvements for the model calibration could be obtained integrating erosion and deposition rates estimated through fallout radionuclides based assessments (Alewell et al., 2014, Porto et al., 2014, Meusburger et al., 2016) or even sediment delivery rates based on marker approaches assessing sediment source attribution in catchments (Alewell et al., 2016). These could be used to test both, the validity of the spatial erosion and sediment transport patterns as well as the calibration quality (Quijano et al., 2016). About 20,000 and 25,000 topsoil samples collected throughout Europe between 2009 and 2012 are currently stored in the facilities of Joint Research Centre. These samples were collected using a harmonised methodology with the purpose of producing statistics on soil characteristics at European level, accompanied by information about their land use history. They could be seen as an opportunity to integrate fallout radionuclide derived soil erosion and deposition rates in large-scale modelling.

## 4 Conclusions

Large-scale net soil loss and deposition modelling connected to sediment transfer and fluxes is crucial to assess holistically the impact of soil degradation across landscapes. Hypotheses such as whether soil erosion processes are net source or sink of carbon can be evaluated, especially when coupled to biogeochemical models estimating CO_2_ fluxes through mineralization or carbon sequestration through burial. Integrating the state-of-the-art environmental parameters of RUSLE2015 in the spatially distributed sediment delivery model WaTEM/SEDEM, we performed a first estimation of the potential net soil and SOC losses by water erosion in Europe. Although our modelling approach presents an important step forward by allowing high resolution large-scale prediction of soil loss (25 × 25 m), supported by good calibration results, the insights gained by the analysis of the results highlight the need to further improve the calibration scheme of the model transport parameter in order to better reconcile the good agreement between predicted and measured sediment yield with the spatial patterns of erosion and deposition. For WaTEM/SEDEM to serve as an effective tool for both ex-ante and ex-post policy evaluations and to increase the current understanding of erosion effects on current carbon budgets, the way forward relies on the introduction of spatially distributed calibration procedures to more effectively capture the changes in transport capacity across the different landscape features. Moreover, future research should be directed towards improving the database of sediment yield (SY) measurements. Beyond the limit related to the number of available data, the results of this study highlight the limit related to the data quality. RUSLE-based models estimates soil loss due to inter‐rill and rill erosion processes. Other geomorphological processes contributing to the catchment sediment yield – for instance, gullying, tillage erosion, bank and channel erosion and re-entrainment of landslide sediments – can be active on the landscape. Therefore, for calibration/validation purposes the use SY data of catchments dominated by interrill and rill process should be preferred.

## References

[bib1] Alatorre L.C., Beguería S., García-Ruiz J.M. (2010). Regional scale modeling of hillslope sediment delivery: a case study in the Barasona Reservoir watershed (Spain) using WATEM/SEDEM. J. Hydrol..

[bib2] Alatorre L.C., Beguería S., Lana-Renault N., Navas A., García-Ruiz J.M. (2012). Soil erosion and sediment delivery in a mountain catchment under scenarios of land use change using a spatially distributed numerical model. Hydrol. Earth Syst. Sci..

[bib3] Alewell C., Meusburger K., Juretzko G., Mabit L., Ketterer M.E. (2014). Suitability of ^239+240^Pu and ^137^Cs as tracers for soil erosion assessment in mountain grasslands. Chemosphere.

[bib4] Alewell C., Birkholz A., Meusburger K., Schindler Wildhaber Y., Mabit L. (2016). Quantitative sediment source attribution with compound-specific isotope analysis in a C3 plant-dominated catchment (central Switzerland). Biogeosciences.

[bib5] Bakker M.M., Govers G., van Doorn A., Quetier F., Chouvardas D., Rounsevell M. (2008). The response of soil erosion and sediment export to land-use change in four areas of Europe: the importance of landscape pattern. Geomorphology.

[bib6] Ballabio C., Panagos P., Monatanarella L. (2016). Mapping topsoil physical properties at European scale using the LUCAS database. Geoderma.

[bib7] Beven K., Binley A. (1992). The future of distributed models: model calibration and uncertainty prediction. Hydrol. Process..

[bib8] Beven K., Binley A. (2014). GLUE: 20 years on. Hydrol. Process..

[bib9] Blasone R.S., Vrugt J.A., Madsen H., Rosbjerg D., Robinson B.A., Zyvoloski G.A. (2008). Generalized likelihood uncertainty estimation (GLUE) using adaptive Markov Chain Monte Carlo sampling. Adv. Water Resour..

[bib10] Borrelli P., Robinson D.A., Fleischer L.R., Lugato E., Ballabio C., Alewell C., Meusburger K., Modugno S., Schütt B., Ferro V., Bagarello V., Van Oost K., Montanarella L., Panagos P. (2017). An assessment of the global impact of 21st century land use change on soil erosion. Nat. Commun..

[bib11] Borrelli P., Märker M., Panagos P., Schütt B. (2014). Modeling soil erosion and river sediment yield for an intermountain drainage basin of the Central Apennines, Italy. Catena.

[bib12] Borrelli P., Paustian K., Panagos P., Jones A., Schütt B., Lugato E. (2016). Effect of good agricultural and environmental conditions on erosion and soil organic carbon balance: a national case study. Land Use Policy.

[bib13] Chappell A., Baldock J., Sanderman J. (2015). The global significance of omitting soil erosion from soil organic carbon cycling schemes. Nat. Clim. Change.

[bib14] Desmet P., Govers G. (1996). A GIS procedure for automatically calculating the USLE LS factor on topographically complex landscape units. J. Soil Water Conserv..

[bib15] De Vente J., Poesen J. (2005). Predicting soil erosion and sediment yield at the basin scale: Scale issues and semi - quantitative models. Earth - Sci. Rev.*.,*.

[bib16] De Vente J., Poesen J., Bazzoffi P., van Rompaey A., Verstraeten G. (2006). Predicting catchment sediment yield in Mediterranean environments: the importance of sediment sources and connectivity in Italian drainage basins. Earth Surf. Process. Landf..

[bib17] Doetterl S., Van Oost K., Six J. (2012). Towards constraining the magnitude of global agricultural sediment and soil organic carbon fluxes. Earth Surf. Process. Landf..

[bib18] EEA-European Environmental Agency, 2016. Data and maps. [Online] URL: 〈http://www.eea.europa.eu/data-and-maps〉 (accessed 08.16).

[bib19] EEA-European Environmental Agency, 2017. European river catchments. [Online] URL: 〈http://www.eea.europa.eu/data-and-maps/figures/european-river-catchments-geographic-view-1〉 (accessed 01.2017).

[bib20] FAO ITPS, 2015. Status of the World’s Soil Resources (SWSR) – Main Report. (Food and Agriculture Organization of the United Nations and Intergovernmental Technical Panel on Soils).

[bib21] Feng X., Wang Y., Chen L., Fu B., Bai G. (2010). Modeling soil erosion and its response to land-use change in hilly catchments of the Chinese Loess Plateau. Geomorphology.

[bib22] Govers G., Poesen J. (1988). Assessment of the interrill and rill contributions to total soil loss from an upland field plot. Geomorphology.

[bib23] Jetten V., Govers G., Hessel R. (2003). Erosion models: quality of spatial predictions. Hydrol. Process..

[bib24] Lal R. (2003). Soil erosion and the global carbon budget. Environ. Int..

[bib25] Lal R. (2004). Soil carbon sequestration impacts on global climate change and food security. Science.

[bib26] Lal R., Pimentel D. (2008). Soil erosion: a carbon sink or source?. Science.

[bib27] Lieskovský J., Kenderessy P. (2014). Modelling the effect of vegetation cover and different tillage practices on soil erosion in vineyards: a case study in Vráble (Slovakia) using WATEM/SEDEM. Land Degrad. Dev..

[bib28] Lugato E., Panagos P., Bampa F., Jones A., Montanarella L. (2014). A new baseline of organic carbon stock in European agricultural soils using a modelling approach. Glob. Change Biol..

[bib29] Lugato E., Paustian K., Panagos P., Jones A., Borrelli P. (2016). Quantifying the erosion effect on current carbon budget of European agricultural soils at high spatial resolution. Glob. Change Biol..

[bib30] Meusburger K., Mabit L., Park J.H., Sandor T., Porto P., Alewell C. (2016). A multi-radionuclide approach to evaluate the suitability of 239 + 240Pu as soil erosion tracer. Sci. Total Environ..

[bib31] Montgomery D.R. (2007). Soil erosion and agricultural sustainability. Proc. Natl. Acad. Sci..

[bib32] Minasny B., Malone B.P., McBratney A.B., Angers D.A., Arrouays D., Chambers A., Field D.J. (2017). Soil carbon 4 per mille. Geoderma.

[bib33] Nash J.E., Sutcliffe J.V. (1970). River flow forecasting through conceptual models part I—A discussion of principles. J. Hydrol..

[bib34] Nadeu E., Gobin A., Fiener P., van Wesemael B., van Oost K. (2015). Modelling the impact of agricultural manage-ment on soil carbon stocks at the regional scale: the role of lateral fluxes. Glob. Biol..

[bib35] Panagos P., Borrelli P., Poesen J., Ballabio C., Lugato E., Meusburger K., Alewell C. (2015). The new assessment of soil loss by water erosion in Europe. Environ. Sci. Policy.

[bib36] Panagos P., Imeson A., Meusburger K., Borrelli P., Poesen J., Alewell C. (2016). Soil conservation in Europe: wish or reality?. Land Degrad. Dev..

[bib37] Paustian K., Lehmann J., Ogle S., Reay D., Robertson G.P., Smith P. (2016). Climate-smart soils. Nature.

[bib38] Porto P., Walling D.E., Alewell C., Callegari G., Mabit L., Mallimo N., Meusburger K., Zehringer M. (2014). Use of a 137Cs re-sampling technique to investigate temporal changes in soil erosion and sediment mobilisation for a small forested catchment in southern Italy. J. Environ. Radioact..

[bib39] Quijano L., Beguería S., Gaspar L., Navas A. (2016). Estimating erosion rates using 137 Cs measurements and WATEM/SEDEM in a Mediterranean cultivated field. Catena.

[bib40] Quinton J.N., Govers G., Van Oost K., Bardgett R.D. (2010). The impact of agricultural soil erosion on biogeochemical cycling. Nat. Geosci..

[bib41] Renard, K., Foster, G., Weesies, G., McCool, D., Yoder, D., 1997. Predicting soil erosion by water: a guide to conservation planning with the Revised Universal Soil Loss Equation (RUSLE). Agricultural Handbook No. 703 404. doi:DC0-16-048938-5 65–100.

[bib42] Runnels C.N. (1995). Environmental degradation in ancient Greece. Sci. Am..

[bib43] Stallard R. (1998). Terrestrial sedimentation and the carbon cycle: coupling weathering and erosion to carbon burial. Glob. Biogeochem. Cycles.

[bib44] USDA, 1975. Sediment sources, yield, and delivery ratios. National Engineering Handbook, Section 3 Sedimentation.

[bib45] Vanmaercke M., Poesen J., Verstraeten G., de Vente J., Ocakoglu F. (2011). Sediment yield in Europe: spatial patterns and scale dependency. Geomorphology.

[bib46] Van Oost K., Govers G., Desmet P. (2000). Evaluating the effects of changes in landscape structure on soil erosion by water and tillage. Landsc. Ecol..

[bib47] Van Oost K., Quine T.A., Govers G., De Gryze S., Six J., Harden J.W., Giraldez J.V. (2007). The impact of agricultural soil erosion on the global carbon cycle. Science.

[bib48] Van Rompaey A.J., Verstraeten G., Van Oost K., Govers G., Poesen J. (2001). Modelling mean annual sediment yield using a distributed approach. Earth Surf. Process. Landf..

[bib49] Van Rompaey A.J., Vieillefont V., Jones R.J.A., Montanarella L., Verstraeten G., Bazzoffi P., Dostal T., Krasa J., de Vente J., Poesen J. (2003).

[bib50] Van Rompaey, A., Krasa, J., Dostal, T., Govers, G., 2003. Modelling sediment supply to rivers and reservoirs in Eastern Europe during and after the collectivisation period. In The Interactions between Sediments and Water. Springer Netherlands, pp. 169–176.

[bib51] Van Rompaey A., Bazzoffi P., Jones R.J., Montanarella L. (2005). Modeling sediment yields in Italian catchments. Geomorphology.

[bib52] Verstraeten G., Oost K., Rompaey A., Poesen J., Govers G. (2002). Evaluating an integrated approach to catchment management to reduce soil loss and sediment pollution through modelling. Soil Use Manag..

[bib53] Verstraeten G. (2006). Regional scale modelling of hillslope sediment delivery with SRTM elevation data. Geomorphology.

[bib54] Vigiak O., Sterk G., Romanowicz R.J., Beven K.J. (2006). A semi-empirical model to assess uncertainty of spatial patterns of erosion. Catena.

[bib55] Zhao G., Mu X., Wen Z., Wang F., Gao P. (2013). Soil erosion, conservation, and eco‐environment changes in the loess plateau of China. Land Degrad. Dev..

[bib56] 4 per 1000 initiative, 2016. [Online] URL: 〈www.http://4p1000.org/〉 (accessed 12.2016).

